# Misrepresentations

**Published:** 1882-06

**Authors:** Ad. Lippe

**Affiliations:** Philadelphia


### MISREPRESENTATIONS.

AD. LIPPE, M. D., PHILADELPHIA.

There was a period when "*Fatal Errors*" were freely advocated by professing homœopathists, but the most prominent of them being exposed in some of the homœopathic journals, an apparent cessation of their promulgation was the result. Of late, a much more dangerous and much more detestable mode of attempted perversion of Homœopathy into eclecticism has been resorted to by professing homœopaths. This new mode of proceeding consists in "*Mis-*

*representations.*" To point them *all* out and expose them as they are presented to the profession in the pretended homœopathic journals, would be an herculean task, and we shall, therefore, only notice the most outrageous.

At the December, 1881, meeting of the Philadelphia County Homœopathic Medical Society, a cleverly-written paper on the treatment of intermittent fever came up for discussion; the discussion was published in the April (1882) number of the *Hahnemannian Monthly* and was opened by the President of the *Hahnemann* Club, a member also of the Faculty of the *Hahnemann* Medical College. The misrepresentations in which this gentleman indulged are (as the only member of the Society who ventured to expose them, calls them) so *outrageous* that it becomes our very unpleasant duty to say a few words about them. In fact, the whole opening speech of the debate is one huge misrepresentation. From first to last the speaker misrepresents not only history, Hahnemann and his work and Homœopathy, but he misrepresents himself as a true homœopath when he claims superior success in treating intermittent fever with Chininum sulph. from the first triturition to massive doses. He says, he follows the precepts of Hahnemann, and prescribes for a totality of symptoms. He individualizes each case and gets all the symptoms, those that are most prominent and those that are least so, the modalities, etc., and having done this, he chooses that which is *the homœopathic* remedy for the case, and finds that this is in most cases, or, at least, in a very large percentage of them, Quinine. He believes that all his success in treating such cases is due to his close adherence to Homœopathy.

*Comments.*—Others, Hahnemann, Bœnninghausen, H. C. Allen and a host of old practitioners have declared time and again, that under the precepts of Hahnemann, professedly adhered to by the learned speaker, they have found China and Quinine to be the truly homœopathic remedy in but a *very small percentage* of cases of intermittent fever. There can be but one logical deduction drawn from the presentation of final results when the same laws were applied for the finding of the homœpathic remedy: when one or more persons find that "in our days," Quinine is the truly homœopathic remedy in almost all cases of intermittent fever, while the founder of the school and a host of his faithful disciples declare it to be but very seldom the curative homœopathic remedy; and that one logical deduction is—that one of two parties "*misrepresents*"

either his conception of, and the mode of practicing, Homœopathy, or his final results.

The learned speaker continues *his* misrepresentations; he says: "Is not Quinine the *Simillimum* to intermittent fever, *par excellence?* By Quinine, here, I mean Sulphate of Quinia, Peruvian Bark, China. Cinchonidia and others of that ilk. These are nearly identical, so far as their pathogenetic or curative effects are concerned."

*Comments.*—The men who have followed the precepts of Hahnemann say that Quinine and China are not identical; that they have common paroxysmal attacks of chill and fever, and both have violent, profuse perspiration, with thirst; that China has no thirst during the chill or during the hot stage, but thirst before the chill and before the hot stage, while Quinine has that *not unimportant* symptom, thirst, during the chill and during the hot stage. The learned speaker will find his misrepresentation of the identity of the China-ilks corrected if he will condescend to take up that most excellent little work on "Homœopathic Therapeutics of Intermittent Fever," by Dr. H. C. Allen, who has clearly differentiated between China and Quinine, on page 81.

The speaker goes on augmenting his misrepresentations when he says: "I have heard quite a good many lectures on Homœopathy, and one of the most frequently repeated statements made on such occasions, was to the effect that Hahnemann, while engaged in translating Cullen's Materia Medica into German, was dissatisfied with the explanation given by Mr. Cullen as to the action of Peruvian Bark in the cure of ague, and that he set himself to experiment with that drug, and found that when taken by a person in good health it produced symptoms very similar to those produced in an attack of ague. This, together with other experiments, led to Hahnemann finding that drugs would cure symptoms similar to those that they were capable of producing, or *similia similibus curantur.* Then Homœopathy may be said to have had its foundation laid on bark. Now, was Hahnemann mistaken in all this? And if he was, is it not possible that he was mistaken in other things just as well, and might not Homœopathy be an error altogether? But my experience proves to me that it is not, for just as Hahnemann found that bark would produce symptoms analogous to those of intermittent fever, I have found that bark, or its alkaloid or alkaloids, will cure genuine malarial intermittent fevers."

*Comments.*—The true history differs essentially from the above clumsy misrepresentation, and we may as well remind the very eloquent misrepresenter that assertions and hearsay testimony are not "evidence." We now give *documentary* evidence to destroy the above misrepresentation. Taking up the second volume of Cullen's *Materia Medica*, we there find under Cinchona the following sentence written by a truthful and brainy man: "*And whilst it (Cinchona) is allowed to be a very safe and very powerful remedy, the only question which remains respecting it is, under what circumstances it may be most properly employed.*" The question was asked in good faith and it implies that in some cases of its employment (and it was then, as now, the boasted specific remedy for intermittent fever), it was a curative remedy, that in other cases it failed just as Quinine fails in a large majority of cases, and if it does fail to cure, it never fails to frequently bring life-lasting misery and harm. Hahnemann, the great philosopher, did answer that question promptly after he had proved Peruvian bark on himself and others; and after he found that the peculiar symptoms, resembling those of intermittent fever, were just such symptoms as had been cured by Cinchona, the deduction forced itself on this "thinker" that these cures were brought about under the law of the similars. Hahnemann continued his labors, and now he is cruelly misrepresented by one of his *pretended* followers as having found out merely that bark would produce symptoms analogous to those of intermittent fever. He found out much more; he solved a question asked by Cullen, and he pointed out to us, who do read his writings, just what the circumstances are under which Cinchona must cure intermittent fever. Every true healer has been made familiar with the characteristic Cinchona and Quinine sick-making, and therefore health-restoring, effects on the human organism. Cullen and his contemporaries found that Cinchona would cure genuine and true malarial intermittent fever; Hahnemann found in what circumstances it may be most properly employed, and if the speaker indulges in a coarse misrepresentation of Hahnemann and of homœopathic history, he may as well indulge in his modest claim to have himself found that Quinine and the other China-ilks will cure genuine malarial intermittent fever. Ignoring wilfully the great discoveries of the founder of our school, who so honestly and diligently worked for the benefit of suffering humanity; ignoring all the characteristic symptoms of Cinchona, which Hahnemann gave the profession; ignoring the very preface to Cinchona written by Hahnemann; ignoring all characteristic symptoms of other well-proved and well-known remedies applicable for the cure of "Intermittent Fever." We are really astonished to see how this *learned* man cunningly misrepresents other remedies. He lays down as one of the characteristic symptoms of Natr. mur.: "Chills commence at 11 o'clock, A. M.," while any tyro in materia medica knows that Natr. mur. shakes at 10 A. M. sharp, and that Baptisia shakes at 11 A. M.

When the gentleman, who shows himself so ignorant of Homœopathy, its literature and its materia medica, was asked in what dose he gave Quinine, his characteristic reply was: "In whatever dose I please or think will be best in the given case. 'Let no pent-up Utica contract our powers. The whole boundless Continent is ours.'" Might he not as well have said (*Hahnemannian Monthly*, p. 215, vol. 4): "I take no stock in medical Popes and Bosses; I do not care a continental for Hahnemann, his observations, his teachings—not I. I believe in eclecticism, and desire to accomplish the perversion of Homœopathy into it. I believe in misrepresentations and in any auxiliary and supplementary means to accomplish this end. *My opinion* is supreme—let us be governed, as heretofore, by opinions, but avoid strict principles which establish 'laws.' No law for us. 'Let no pent-up Utica contract our powers.'" Our limited space does not permit us to dwell on the misrepresentations, bad logic and hard assertions developed in that "discussion;" in fact, we have served up enough of this unsavory dish to make the patient reader of these lines heart-sick and disgusted. Patience! Just let these men keep on uttering absurdities and Misrepresentations! Somebody will, in the near future, say—enough!

